# Pied de Madura négligé: un défi thérapeutique

**DOI:** 10.11604/pamj.2014.18.295.4949

**Published:** 2014-08-14

**Authors:** Nada El Moussaoui, Badredine Hassam

**Affiliations:** 1 Service de Dermatologie, CHU Ibn Sina, Université Med V, Souissi, Rabat, Maroc

**Keywords:** Mycétome, pied de Madura, tuméfaction, Mycetoma, Madura foot, swelling

## Image en medicine

Le mycétome ou pied de Madura est une maladie granulomateuse chronique, se développant dans le tissu mou sous-cutané et peuvent atteindre l'os faisant toute la gravité de l'affection. Il touche classiquement les sujets d'origine rurale, en particulier les agriculteurs. L'inoculation suit généralement un traumatisme mineur par épines ou échardes ou instruments souillés, principalement au niveau du pied. La triade caractéristique de la maladie comprend une tuméfaction, fistulisation de l'abcès et émission de grains colorés de nature fongique (eumycétome) ou bactérienne (actinomycétome). La confirmation mycologique et histologique est nécessaire. L'exploration radiologique devrait être systématique afin de rechercher des lésions osseuses et conditionner la conduite thérapeutique. Bien que les deux infections se manifestent semblablement, les actinomycétomes évoluent favorablement sous antibiotiques, alors que les eumycétomes nécessitent une excision chirurgicale en plus des antifongiques. Les complications peuvent mettre en jeu le pronostic fonctionnel (amputation) voire vital (décès par septicémie secondaire). Nous rapportons le cas de Mme H.E, 53 ans, ouvrière de chantier, qui consulte pour une tumeur polyfistulisée du pied gauche survenant il y a 10 ans suite à un traumatisme local. La radiographie du pied gauche ne révèle pas d'anomalie osseuse. La culture sur milieux de Sabouraud à 37°C isole Madurella Mycetomatis. La patiente est mise sous Terbinafine pendant 6 mois sans aucune amélioration, puis adressée en chirurgie plastique pour chirurgie de réduction.

**Figure 1:**
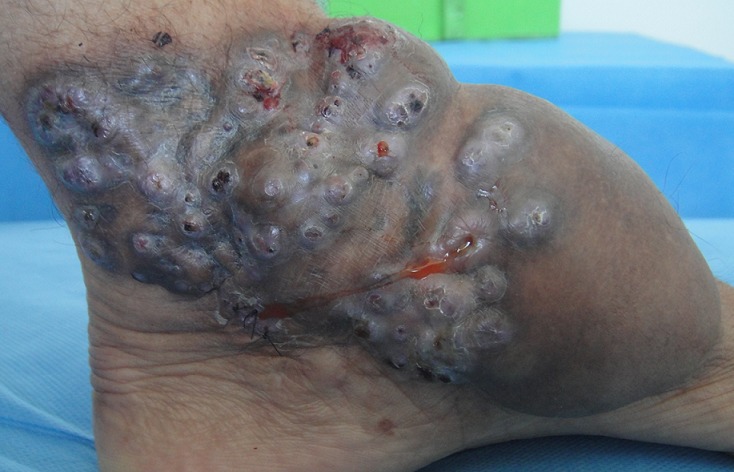
Tumeur polylobée polyfistulisée

